# Conserved Lysine Acetylation within the Microtubule-Binding Domain Regulates MAP2/Tau Family Members

**DOI:** 10.1371/journal.pone.0168913

**Published:** 2016-12-21

**Authors:** Andrew W. Hwang, Hanna Trzeciakiewicz, Dave Friedmann, Chao-Xing Yuan, Ronen Marmorstein, Virginia M. Y. Lee, Todd J. Cohen

**Affiliations:** 1 Department of Pathology and Laboratory Medicine, Perelman School of Medicine, University of Pennsylvania, Philadelphia, Pennsylvania, United States of America; 2 Department of Biochemistry and Biophysics, University of North Carolina, Chapel Hill, North Carolina, United States of America; 3 Department of Biochemistry and Biophysics, Abramson Family Cancer Research Institute, Perelman School of Medicine, University of Pennsylvania, Philadelphia, Pennsylvania, United States of America; 4 Alexion Pharmaceuticals Inc, New Haven, Connecticut, United States of America; 5 Department of Neurology, UNC Neuroscience Center, University of North Carolina, Chapel Hill, North Carolina, United States of America; Centre Hospitalier de l'Universite Laval, CANADA

## Abstract

Lysine acetylation has emerged as a dominant post-translational modification (PTM) regulating tau proteins in Alzheimer’s disease (AD) and related tauopathies. Mass spectrometry studies indicate that tau acetylation sites cluster within the microtubule-binding region (MTBR), a region that is highly conserved among tau, MAP2, and MAP4 family members, implying that acetylation could represent a conserved regulatory mechanism for MAPs beyond tau. Here, we combined mass spectrometry, biochemical assays, and cell-based approaches to demonstrate that the tau family members MAP2 and MAP4 are also subject to reversible acetylation. We identify a cluster of lysines in the MAP2 and MAP4 MTBR that undergo CBP-catalyzed acetylation, many of which are conserved in tau. Similar to tau, MAP2 acetylation can occur in a cysteine-dependent auto-regulatory manner in the presence of acetyl-CoA. Furthermore, tubulin reduced MAP2 acetylation, suggesting tubulin binding dictates MAP acetylation status. Taken together, these results uncover a striking conservation of MAP2/Tau family post-translational modifications that could expand our understanding of the dynamic mechanisms regulating microtubules.

## Introduction

The microtubule (MT)-associated protein (MAP) tau is expressed primarily in the nervous system, in which six tau isoforms exist containing either three (3R-tau) or four (4R-tau) MT-binding repeat domains. Along with the flanking regions, the MT-binding domains coordinate tau-MT binding and stabilization [1, 2]. Over the last several years, we and others demonstrated that tau is subject to lysine acetylation mainly within the MT-binding repeat region (MTBR), identifying a new and emerging post-translational modification (PTM) that could dominantly regulate multiple aspects of tau function [3–7]. Indeed, our previous *in vitro* experiments showed that tau acetylation is sufficient to impair normal tau-MT interactions, prevent tau-mediated stabilization of MTs, and promote the formation of tau fibrils that resemble those observed in diseased brain [3].

Neuropathological analysis of human brain tissue showed that acetylated tau at residue K280 (Lys280) within the tau MTBR accumulated in tauopathy brains and produced a distinctly pathological signature in Alzheimer’s disease (AD) and also a panel of other tauopathies including corticobasal degeneration (CBD), progressive supranuclear palsy (PSP), and several FTDP-17 familial dementia patients. K280-acetylated tau was not readily detectable in control brain tissue or cultured wild-type cells or neurons, thus correlating tau acetylation at K280 with the onset or progression of tauopathy [8, 9]. Recently, however, more sensitive mass spectrometry studies indicated that tau is normally acetylated in wild-type mouse brain at several lysines spanning the MTBR, suggesting a normal physiological role for tau acetylation in regulating MT-binding or MT stabilization [7]. Supporting this possibility, early biochemical studies showed that positively charged tau residues K280 and K281 directly contact MTs, strongly implicating this double lysine containing PHF6* motif (^275^VQIINK/K^281^) as a critical hotspot for tau-MT binding in addition to facilitating conformational β-structure and tau aggregation [10]. Either genetic deletion of residue K280 (ΔK280), as observed in familial FTDP-17 dementia [11], or lysine acetylation on residue K280, as observed in AD and related tauopathies [3], may be sufficient to neutralize the positive charge within this region, thereby impairing tau-MT interactions. The homologous lysine residue residing in the 3^rd^ MTBR repeat, K311 within the PHF6 motif (^306^VQIVYK^311^), has also been shown to regulate tau biochemical properties, as a variety of substitutions at residue K311 alter tau aggregation kinetics [12]. Thus, MTBR lysines likely engage in critical electrostatic charge interactions; these are the same lysine residues that are also subject to acetylation, which functionally regulates tau-MT binding and stabilizing activity. Whether acetylation-dependent mechanisms similarly regulate MAPs beyond tau has not been investigated.

The abundance of tau acetylation sites in normal wild-type mice supports a broader physiological role for MAP acetylation in cytoskeletal dynamics [7]. Tau is highly homologous to the related MAP2 and MAP4 proteins [13, 14], which possess 3 or 4 MTBRs with highly conserved lysine-rich repeats. While global MAP acetylation *per se* has not been characterized, evidence supports an emerging network of acetylated factors localized to the cytoskeleton and also associated with MTs [15]. Moreover, components of the acetylation machinery are particularly enriched in close proximity to MTs including acetyltransferases such as the Elongator complex [16] and tubulin acetyltransferse (TAT) [17] as well as the deacetylases HDAC6 [18] and SIRT2 [19]. In this study, we describe lysine acetylation as a novel conserved regulatory mechanism among MAP2/Tau family members, which has implications for the regulation of MAP function and microtubule dynamics.

## Materials and Methods

### Plasmids and Cell Culture

The human tau isoform containing 2 N-terminal inserts and 4 MTBR regions (2N4R) was cloned into pCDNA5/TO vector (Invitrogen). A rat MAP2c construct containing an HA-tag was kindly provided by Dr. Shelley Halpain (University of California, San Diego) and a human MAP4-GFP plasmid was kindly provided by Dr. Chloe Bulinski (Columbia University). A plasmid containing wild-type CBP or the inactive L1435A/D1436A mutant (pcDNA3.1-CBP and CBP-LD) was kindly provided by Dr. Tso-Pang Yao (Duke University). All described plasmids were transfected into QBI-293 cells using Fugene-6 (Promega) or Lipofectamine 2000 (Thermofisher) reagents per the manufacturers protocols. Transfected QBI-293 cells were evaluated for MT bundling by immunofluorescence microscopy using anti-tau/MAP2 antibodies (polyclonal anti-tau, Dako), anti-MAP4-GFP (polyclonal anti-GFP, Santa Cruz), and anti-acetylated tubulin antibodies (Sigma).

### Recombinant *in vitro* methods

#### Purified MAP protein preparations

Tau-K18 (2N4R-tau spanning residues 244–372), MAP2c fragments (spanning residues 280–398 of the full-length 467 residue rat MAP2c), and MAP4 fragments (spanning residues 925–1102 of full-length 1152 residue human MAP4) were purified using an AKTA-Pure FPLC chromatography system (GE). The proteins were expressed in Escherichia coli BL21(DE3) RIL strain. Bacteria were grown in LB media containing ampicillin at 37°C and induced with isopropyl-D -thiogalactopyranoside at a final concentration of 0.8 mM when the OD reached 0.6. After agitation for 2 h, cells were harvested by centrifugation. The pellet was re-suspended in high-salt RAB buffer [0.1 M MES, 1 mM EGTA, 0.5 mM MgSO_4_, 0.75 M NaCl, 0.02 M NaF, 0.1 mM PMSF, 0.1% protease inhibitor cocktail (100 μg/mL each of pepstatin A, leupeptin, TPCK, TLCK, and soybean trypsin inhibitor and 100 mM EDTA), pH 7.0] and homogenized. The cell lysates were heated to 100°C for 10 min, rapidly cooled on ice for 20 min and centrifuged at 70,000xg for 30 min. Supernatants were dialyzed into FPLC buffer A [20 mM piperazine-N, N-bis(2-ethanesulfonic acid) pH 6.5, 10 mM NaCl, 1 mM EGTA, 1 mM MgSO_4_, 2 mM DTT, 0.1 mM PMSF], applied onto a HiTrap Sepharose HP IEX cation-exchange column (GE Healthcare), and eluted with a 0–0.4 M NaCl gradient using an AKTA-Pure FPLC system (GE Healthcare). The fractions were checked for the presence of the tau proteins by sodium dodecyl sulfate-polyacrylamide gel electrophoresis (SDS- PAGE) followed by Coomassie Blue R-250 staining. The fractions containing enriched tau or MAP2c were pooled together and dialyzed against 100 mM sodium acetate buffer, pH 7.0. Importantly, all purified proteins were subject to a critical heat-denaturation step at 100°C to inactivate any endogenous bacterial enzymes and simultaneously enrich for heat-stable tau proteins. Proteins were concentrated using Amicon Ultra centrifugal filter units (Millipore Corporation, Billerica, MA), and protein concentrations were determined using the bicinchoninic acid protein assay (Pierce, Rockford, IL) with bovine serum albumin as the protein standard.

#### In vitro acetylation reactions

Tau, MAP2c, or MAP4 fragments (5 μM) were either mock acetylated with coenzyme A alone or modified *in vitro* using acetyl-CoA (Sigma) in acetylation reaction buffer (50 mM Tris–HCl pH 8.0, 10% glycerol, 1 mM DTT, 100 μM EDTA, 1 mM PMSF, 0.4 mM acetyl-CoA cofactor) at 37°C for 1hr. For radiolabeling experiments, 50 nCi [^14^C]-labeled acetyl-CoA (Perkin Elmer) was substituted for unlabeled acetyl-CoA. Reaction products were analyzed by resolving proteins on 15% SDS-PAGE gels followed by transfer to nitrocellulose membranes and phosphorimaging analysis using Storm software, or immunoblotting with the indicated antibodies. MAP-rich fractions containing bovine brain-derived MAPs were purchased from Cytoskeleton and desalted, where indicated, using Zeba Spin Desalting Columns (Thermofisher) prior to acetylation reactions. For tubulin competition experiments, purified brain tubulin (Cytoskeleton) was titrated into acetylation reactions at final concentration of 1–10 μM and preincubated for 10 min at 37°C prior to initiation of acetylation by addition of acetyl-CoA. All reactions were performed with a minimum of N = 3 independent experimental replicates.

For filter-binding assays, reactions containing tau-K18, MAP2c, or MAP4 fragments were initiated by the addition of [^3^H]-acetyl-CoA (2.35 Ci mmol^-1^, Perkin Elmer) at concentrations ranging from 2.5 μM to 1 mM acetyl-CoA, incubated at 37° C, and allowed to proceed for 1 hr. Reactions were stopped by the addition of 10 μl of 3 mM CoA, and 25 μl of the reaction was then spotted to a P81 filter paper (Whatman), washed in 10 mM HEPES pH 7.5 three times, and dried using acetone. Incorporated [^3^H]-acetyl-CoA was then measured using the Perkin Elmer Tri-Carb 2800TR Liquid Scintillation Analyzer, and the molar amount of acetyl groups incorporated were calculated from a standard curve of the radiolabeled acetyl-CoA.

### Biochemical analysis of mouse brain

This study was carried out in accordance with the University of North Carolina (UNC) Institutional Animal Care and Use Committee (IACUC). The protocol was approved by the UNC IACUC Committee (ID# Number: 14.107.0). Mice were anesthetized with isoflurane or a ketamine/xylazine mixture and all efforts were made to minimize pain suffering. Whole cortex from wild-type (WT) and tau knock-out (KO) mice were high-salt extracted, boiled where indicated, and resulting homogenates were incubated with acetyl-CoA containing reaction buffers. For acetyltransferase assays using brain lysates, boiled mouse lysates or salt-extracted human lysates were dialyzed into PBS and supplemented with Tris pH 8.0 to a final concentration of 50 mM.

### Biochemical extraction of cultured cells

Cells from 6-well culture dishes were scraped into 300 μl RIPA buffer (50 mM Tris pH 8.0, 150 mM NaCl, 1% NP-40, 5 mM EDTA, 0.5% sodium deoxycholate, 0.1% SDS) containing 1 mM phenylmethylsulfonyl fluoride (PMSF), a mixture of protease inhibitors (1 μM pepstatin, leupeptin, *N*-p-Tosyl-l-phenylalanine chloromethyl ketone, Nα-Tosyl-l-lysine chloromethyl ketone hydrochloride, trypsin inhibitor; Sigma), and a mixture of phosphatase inhibitors (2 mM imidazole, 1 mM NaF, 1 mM sodium orthovanadate; Sigma). Cell homogenates were sonicated and centrifuged at 100,000xg for 30 min at 4^°^C to provide a RIPA soluble fraction. Soluble lysates were analyzed by immunoblotting using the described antibodies. The antibodies used for western analysis were as follows: polyclonal anti-acetylated-lysine (1:1000, Cell Signaling #9441), anti-tau C-terminal clone T46 (1:1000, Thermofisher), and anti-acetylated tau, Ac-K280 [3] (1:1000), polyclonal anti-GFP (1:1000, Santa Cruz).

### Cell-based immunoprecipitation/acetylation reactions

Acetyltransferase assays were performed by immunoprecipitating tau or MAP2c proteins (using anti-tau/MAP2 antibody, clone T46) (Thermofisher) or MAP4 (using polyclonal anti-GFP) (Santa Cruz). MAPs were bound by protein A/G beads followed by immunoblotting using polyclonal anti-acetyl-lysine antibodies (Cell Signaling) or alternatively radiolabeling was performed by addition of [^14^C]-Acetyl-CoA (Perkin Elmer) for 2 hr at 37°C. Radiolabeled acetylated MAPs bound to beads were eluted and analyzed by SDS-PAGE and Coomassie staining followed by phosphorimaging using Storm software. For acetyltransferase analysis using WT and tau KO brain lysates, boiled high-salt extracted mouse cortical lysates were dialyzed into PBS and supplemented to a final concentration of 50 mM Tris pH 8.0. The desired cofactors, either CoA or acetyl-CoA, were supplemented to 1 mM and incubated for 2 hr at 37^°^C followed by immunoblotting using the indicated antibodies.

### Mass spectrometry

NanoLC nanospray MS-MS analysis was performed at the University of Pennsylvania proteomics core facility, which identified acetylated MAP2c and MAP4 lysine residues (Tables 1 and 2). MAP proteins were immunoprecipitated with T46 (MAP2c) or GFP (MAP4) antibodies and separated by SDS-PAGE followed by gel excision and mass spectrometry analysis using LTQ XL* Linear Ion Trap Mass Spectrometer (Thermo Scientific). Data was acquired using Xcalibur software (Thermo Scientific) and analyzed using Mascot, Scaffold, and/or PEAKS software programs. Significantly acetylated peptide cutoffs showed p-values < 0.05 and peptide scores > 20.

**Table 1:** Acetylated lysines identified in MAP2c

Site	MTBR	p-value	Peptide
K107	N	9.40E-05	IVQVVTAEAVAVL**K**GEQEKEAQHK
K230	N	1.00E-07	AG**K**SGTSTPTTPGSTAITPGTPPSYSSR
K273	N	0.0025	TPGTP**K**SGILVPSEK
K273, K282	N	2.70E-05	TPGTP**K**SGILVPSE**K**K
K273, K283	N	3.90E-05	TPGTP**K**SGILVPSEK**K**
K282	N	0.024	SGILVPSE**K**K
K283	N	0.057	**K**VAIIR
K282, K283	N	2.60E-06	SGILVPSE**KK**VAIIR
K292	N	0.0065	TPP**K**SPATPK
K292, K298	N	0.068	TPP**K**SPATP**K**QLR
K298	N	0.022	SPATP**K**QLR
K311	Y	0.0013	LINQPLPDL**K**NVK
K314, K316	Y	0.00032	NV**K**S**K**IGSTDNIK
K316, K324	Y	5.40E-06	S**K**IGSTDNI**K**YQPK
K324	Y	0.00075	IGSTDNI**K**YQPK
K328	Y	0.00029	YQP**K**GGQVQIVTK
K328, K337	Y	6.20E-06	YQP**K**GGQVQIVT**K**K
K328, K338	Y	0.07	YQP**K**GGQVQIVTK**K**
K337	Y	5.00E-05	GGQVQIVT**K**K
K337, K338	Y	7.10E-07	GGQVQIVT**KK**IDLSHVTSK
K338	Y	3.60E-05	**K**IDLSHVTSK
K347	Y	0.0011	IDLSHVTS**K**CGSLK
K347, K352	Y	3.80E-05	IDLSHVTS**K**CGSL**K**NIR
K364, K369	Y	0.0092	V**K**IESV**K**LDFK
K369	Y	0.0024	VKIESV**K**LDFK
K379	Y	0.00055	AQA**K**VGSLDNAHHVPGGGNVK
K396	Y	2.50E-05	VGSLDNAHHVPGGGNV**K**IDSQK
K401	Y	7.90E-06	IDSQ**K**LNFR

MAP2c acetylated lysines identified by mass spectrometry are listed by residue number using the 467 amino acid rat MAP2c sequence. The location of the identified lysine within the MTBR (Yes/No), p-value for each peptide, and peptide sequence containing the primary acetylated lysine in bold are shown.

**Table 2:** Acetylated lysines identified in MAP4

Site	MTBR	p-value	peptide
K702	N	0.065	T**K**PLATTQPAK
K718	N	0.023	A**K**TQPTSLPK
K838, K847	N	0.0018	KPTAI**K**TEGKPAEV**K**K
K852	N	0.015	MTA**K**SVPADLSRPK
K871	N	0.00012	**K**TTTLSGTAPAAGVVPSR
K912	N	0.0074	KPTSA**K**PSSTTPR
K933	Y	0.0042	LATNTSAPDL**K**NVR
K938	Y	0.076	S**K**VGSTENIK
K997, K998	Y	0.05	VQIVS**KK**VSYSHIQSK
K1028	Y	0.019	HVPGGGNVQIQN**K**K
K1034	Y	0.00048	KVDIS**K**VSSK
K1060	Y	0.002	IESQ**K**LNFK
K1070	Y	0.031	AQA**K**VGSLDNVGHLPAGGAVK

MAP4 acetylated lysines identified by mass spectrometry are listed by residue number using the 1152 amino acid longest human MAP4 sequence. The location of the identified lysine within the MTBR (Yes/No), p-value for each peptide, and peptide sequence containing the primary acetylated lysine in bold are shown.

## Results

### MAP2/Tau family members are subject to lysine acetylation

**Fig 1:**
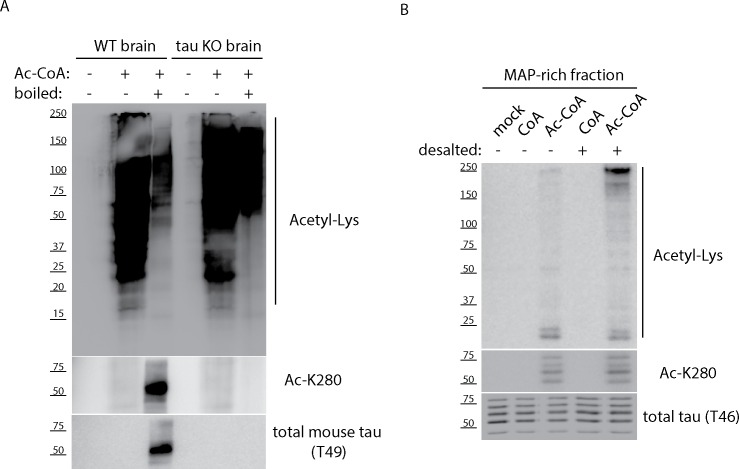
MAP-rich fractions and mouse brain lysates contain targets of acetylation. **A)** Cortical brain tissue from wild-type (WT) and tau knock-out (KO) mice were high-salt extracted, boiled where indicated, and resulting homogenates were incubated with acetyl-CoA containing reaction buffers. Samples were analyzed by immunoblotting using anti-acetyl-lysine, Ac-K280, and total tau (T49) antibodies. **B)** MAP-rich fractions from bovine brain were desalted, where indicated, and incubated with CoA or acetyl-CoA followed by immunoblotting similar to **(A)** above. Shown are representative immunoblot analysis from N = 3 independent experiments.

In order to investigate MAP acetylation, we incubated mouse brain lysates (Fig 1A) or MAP-rich fractions purified from bovine brain (Fig 1B) with acetyl-CoA in an *in vitro* acetylation reaction. In both instances, we observed accumulation of acetylated target proteins ranging from ~25–250 kDa even in the complete absence of tau proteins (~50–65 kDa) using tau KO brain lysates, suggesting that MAPs in addition to tau are potentially subject to acetylation. Furthermore, the ~250 kDa MAP2 band was strongly detected with the anti-acetyl-lysine antibody in MAP-rich fractions incubated with acetyl-CoA and was more prominently observed upon desalting of MAP fractions, which led to increased acetylation efficiency (Fig 1B).

**Fig 2:**
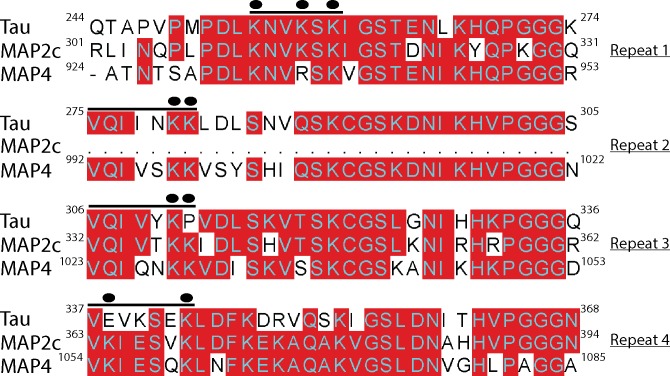
Tau, MAP2c, and MAP4 sequence alignments illustrate extensive MTBR homology and conservation of critical lysines The microtubule-binding repeats 1–4 from tau, MAP2c, and MAP4 were aligned and exact amino acid homology among the MAPs is depicted with red shading. Solid black lines identify two putative MAP acetylation motifs (see Tables 1 and 2 for all identified acetylated residues), and specific acetylated lysines that are enriched within the identified motifs are highlighted above the indicated lysine residues with filled black circles.

To further evaluate MAP acetylation, we focused on MAP2c and MAP4. MAP2c is the shortest MAP2 isoform that is amenable to molecular and biochemical analysis by virtue of its lower molecular weight (~65–75 kDa) compared to the much larger full-length MAP2 isoform (~250 kDa). Alignment of the MTBR domains of tau, MAP2c, and MAP4 highlights the extensive ~75% homology within their functional repeats (Fig 2) [20]. All three MAPs possess highly lysine-rich MTBRs that mediate MAP-MT electrostatic interactions, indicating the potential for conserved acetylation within their repeats. Notably, tau/MAP4 alignments showed conservation of critical tau residues K280/K281 within the 2^nd^ repeat motif ^275^VQIINK/K^281^, and the combined tau/MAP2c/MAP4 alignment showed conservation of the 3^rd^ repeat motif harboring tau lysine K311, ^306^VQIVYK^311^ (Fig 2).

**Fig 3:**
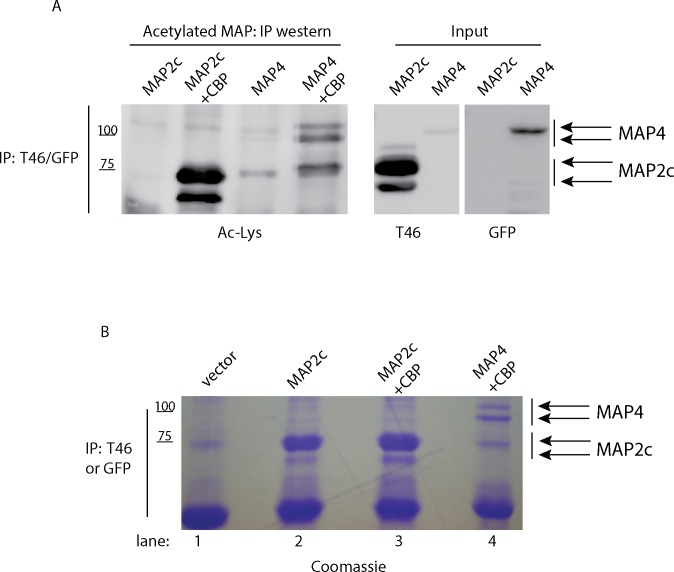
MAP2 and MAP4 are subject to reversible lysine acetylation **A)** QBI-293 cells were co-transfected with MAP2c or MAP4-GFP expression plasmids in the presence of CBP to promote full acetylation, followed by immunoprecipitation and immunoblot analysis using acetylated lysine, T46 or GFP antibodies. The monoclonal antibody T46 detects a highly conserved C-terminal epitope that is identical in tau and MAP2c proteins. **B)** Immunoprecipitation reactions from cells transfected with vector alone (lane 1), MAP2c alone (lane 2), MAP2c and CBP (lane 3), or MAP4 and CBP (lane 4) were performed with T46 or GFP antibodies. Samples were separated by SDS-PAGE and Coomassie stained followed by gel band excision and analysis by mass spectrometry (NanoLC nanospray MS-MS, see methods). In the absence of CBP, no lysine acetylation was detected. In the presence of CBP, MAP2c and MAP4 acetylation sites were identified and full peptide details are listed in Tables 1 and 2. Shown are representative immunoblots from N = 3 independent experiments. Coomassie staining, gel band excision, and mass spectrometry was performed in triplicate confirming the identity of acetylated lysine residues from three independent MAP acetylation experiments.

MAP2c and MAP4 acetylation was assessed by co-expression with the constitutively active acetyltransferase CBP, previously shown to acetylate tau isoforms with high affinity using *in vitro* and cell-based assays [3, 6, 21]. QBI-293 cells were employed for MAP acetylation experiments since these cells have low levels of tau/MAP2 and are therefore amenable to MAP expression studies without confounding levels of endogenous MAPs. MAP2c or MAP4 proteins were immunoprecipitated from co-transfected cells, and MAP acetylation was detected using an anti-acetyl-lysine antibody (Fig 3A). While MAP acetylation was undetectable in the absence of CBP, co-expression with CBP led to the accumulation of acetylated MAP2c and MAP4, which migrated as a series of acetylated protein bands ~ 65 kDa (MAP2c) and ~100 kDa (MAP4).

To identify acetylated lysine residues, large-scale transfections in the presence of CBP were used to immunopurify acetylated MAP2c and MAP4 proteins, yielding highly purified MAPs, as determined by Coomassie staining of eluted fractions. Approximately 5–10 μg of total purified MAPs were isolated from cell extracts (Fig 3B). Our MAP enrichment strategy enabled highly sensitive detection of lysine acetylation by mass spectrometry analysis upon gel excision and subsequent acetylated peptide mapping using nanoLC/nanospray/MS/MS. We note that incorporation of the deacetylase inhibitors trichostatin A (TSA) and nicotinamide (NCA), which block HDAC/sirtuin family members, was critical to preserve MAP acetylation status throughout the MAP extraction and isolation process.

Acetylated lysine-containing peptides were identified for both MAP2c (Table 1 and S1 Fig) and MAP4 (Table 2), many of which clustered within the MTBR regions similar to tau proteins (Fig 2). This observation was despite the fact that lysine residues are distributed throughout MAP2c and MAP4, suggesting specificity for acetylation within the MTBR. We observed several regions within the MTBRs of MAP2c and MAP4 that represent putative acetylation motifs (Fig 2, see motifs highlighted by solid black lines). In repeat 1, we identified a conserved lysine-rich cluster [**K**-N-V-**K**/R-S-**K**] in which either 2 or 3 lysines were acetylated in both MAP2c and MAP4. Further acetylation site comparisons identified a more general consensus motif [V-Q/**K**-I-X-X-X/**K**-**K**] present at the N-terminus of MAP2c repeats 3/4 and MAP4 repeats 2/3/4, in which several acetylated lysines were enriched. Consistent with these two regions acting as putative MAP acetylation motifs, tau proteins are similarly acetylated in repeat 1 at residue K259 [^254^K-N-V-K-S-**K**^259^] [4], repeat 2 at residues K280 and K281 [^275^V-Q-I-I-N-**K**-**K**^281^] [3, 22], and repeat 3 at residue K311 (^306^V-Q-I-V-Y-**K**^311^) [3]. Thus, two distinct lysine-rich motifs within the MTBR emerged as conserved hot-spots that are particularly susceptible to acetylation, possibly due to distinct conformation and/or increased lysine accessibility within these regions.

### MAP acetylation can occur via an auto-catalytic mechanism

**Fig 4:**
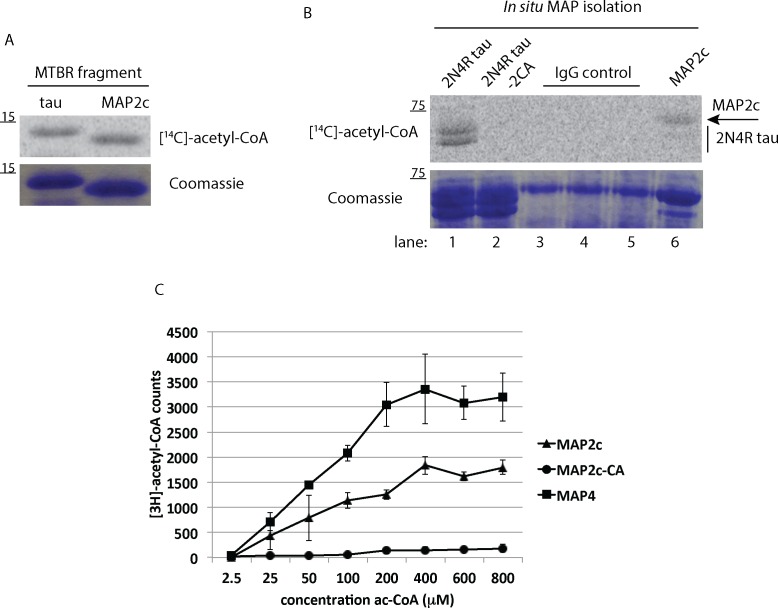
MAPs are subject to auto-acetylation within the MTBR domain **A)** Recombinant tau-K18 (residues 244–372) or MAP2c (residues 280–388) fragments were incubated in acetylation reactions containing [^14^C]-labeled acetyl-CoA followed by SDS-PAGE and phosphorimaging using Storm software to detect radiolabeled MAPs. **B)** Immunoprecipitated 2N4R tau (lane 1), 2N4R-2CA mutant tau (lane 2), or MAP2c proteins (lane 6) derived from QBI-293 lysates were immobilized on agarose beads, incubated with [^14^C]-Acetyl-CoA, and analyzed by SDS-PAGE and Coomassie staining followed by phosphorimaging analysis. Replicate IgG control samples (lanes 3–5) were used to clearly separate signal intensities from tau and MAP2c sample lanes. **C)** Purified MTBR fragments from wild-type MAP2c (280–388), a comparable MAP2c fragment containing a cysteine→alanine substitution (C348A), or MAP4 (925–1102) were incubated in acetylation reactions and direct incorporation of radiolabeled acetyl groups was quantified using a liquid scintillation analyzer. Shown are representative analysis using N = 3 technical replicates from N = 3 independent experiments.

While previous studies have suggested that tau acetylation occurs *in trans* by CBP/p300 [3, 5, 6, 21], evidence also indicates that tau auto-acetylation occurs upon incubation of tau proteins with acetyl-CoA alone [21]. Thus, to further confirm MAP acetylation and to determine if the recently described tau auto-acetylation mechanism also applies to MAP2, a truncated MAP2c fragment encompassing the MAP2 MTBR (residues 280–398) was generated and analyzed by *in vitro* acetylation reactions with radiolabeled [^14^C]-acetyl-CoA. Tau and MAP2c acetylation reactions were separated by SDS-PAGE gel, transferred to nitrocellulose membranes, and analyzed by autoradiography and phosphorimaging. In agreement with our previous study [21], the tau-K18 fragment comprising the 4-repeat tau region (residues 244–372), showed robust auto-acetylation, an effect that was similarly observed with the homologous MAP2c MTBR fragment (Fig 4A).

To independently confirm MAP2c auto-acetylation, we isolated transfected tau or MAP2c using immunoprecipitation pull-downs and performed auto-acetylation reactions on MAPs bound to affinity beads, which showed that isolated full-length tau (2N4R) and MAP2c are both capable of self-acetylation upon incubation with acetyl-CoA (Fig 4B). As a negative control, a cysteine-less tau mutant (2N4R-2CA) that impairs auto-acetylation showed no detectable radiolabeling [21]. Finally, direct incorporation of [^3^H]-acetyl groups onto MAP2c and MAP4 lysine residues was measured by quantification of radiolabeled fragments using a filter-binding assay that traps low-molecular weight MAP fragments but not free unconjugated [^3^H]-acetyl-CoA. MAP2c and MAP4 fragments that span the MTBR showed significant acetylation, which increased linearly as a function of increasing concentration of acetyl-CoA from 2.5–800 μM (Fig 4C). The additional 2^nd^ MTBR repeat present in MAP4, but not MAP2c, may account for the increased MAP4 acetylation observed at most acetyl-CoA concentrations. Notably, mutation of the single MAP2c cysteine residue to an alanine (C348A) abrogated acetylation, strongly supporting cysteine-mediated auto-acetylation [21]. Together, these data suggest that CBP-catalyzed acetylation and chemical auto-acetylation are conserved among MAP2/Tau family proteins.

### MAP-Tubulin interactions are regulated by MAP acetylation

**Fig 5:**
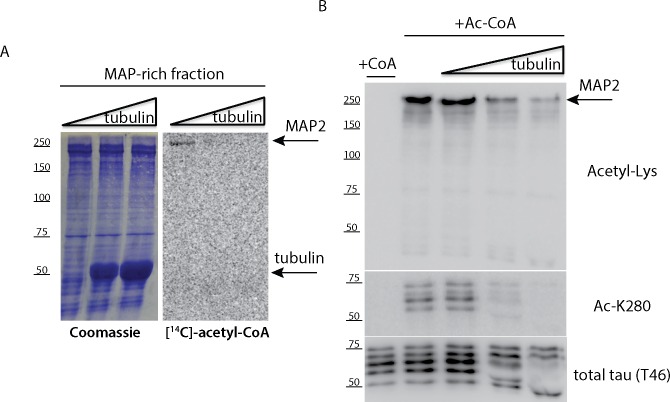
MAP interactions with tubulin impair MAP acetylation **A)** Bovine brain derived MAP-rich fractions were incubated in the absence or presence of increasing concentrations of tubulin (1–10 μM), and analyzed in auto-acetylation reactions containing [^14^C]-acetyl-CoA. **B)** Reactions similar to **(A)** above were incubated with unlabeled acetyl-CoA in the presence of tubulin and analyzed by immunoblotting using acetylated lysine (Acetyl-Lys), acetylated tau (Ac-K280), and total tau (T46) antibodies. We note that the addition of tubulin progressively inhibited tau and MAP2 acetylation. Shown are representative gels and immunoblots from N = 3 independent experiments.

One primary function of MAPs is to bind and stabilize MTs [13], mediated partly via MTBR lysines that associate with MTs [10]. The presence of tubulin is expected to engage MAP lysine residues and therefore limit lysine accessibility required for acetyl group transfer. Thus, we asked whether MAP acetylation still occurred in the presence of increasing concentrations of tubulin. Purified MAP-rich brain fractions containing full-length MAP2 (250 kDa isoform) were incubated with purified tubulin, which led to progressively reduced MAP2 acetylation detected by [^14^C]-acetyl-CoA labeling (Fig 5A). Similarly, immunoblotting with anti-acetylated lysine antibodies showed reduced MAP2 acetylation and a simultaneous reduction of tau acetylation at residue K280 using the acetylated tau-specific antibody ac-K280 (Fig 5B). Therefore, MAP acetylation is negatively regulated by interactions with tubulin that engage positively charged MAP lysine residues.

**Fig 6:**
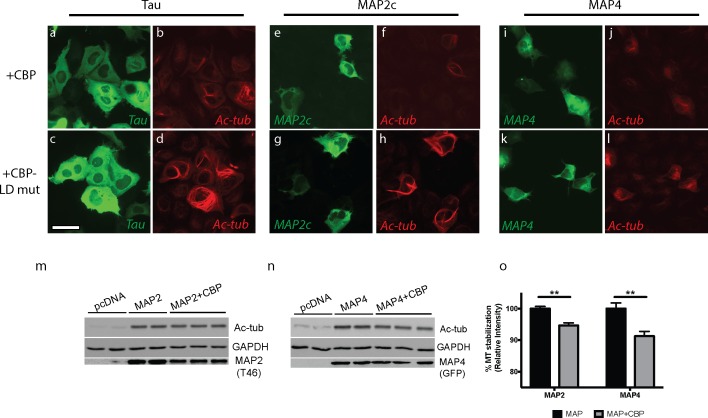
MAP acetylation alters MAP-mediated MT stabilization QBI-293 cells were transfected with 2N4R-tau (panels **a-d**), MAP2c (panels **e-h**), or MAP4 (panels **i-l**) in the presence of wild-type CBP (top row) or an inactive CBP-LD mutant (bottom row). MT bundling in MAP-transfected cells (green) was determined 48 hr later by acetylated tubulin immunofluorescence (red), an indicator of MAP-induced MT stabilization. Note, all MAPs tested promoted baseline MT bundling in the absence of CBP activity (CBP-LD mutant), which was less elaborated upon MAP acetylation with active wild-type CBP. Scale bar represents 50 μm. **m-o)** QBI-293 cells were transfected with MAP2c **(m)** or MAP4 **(n)** in the presence or absence of wild-type CBP and analyzed by immunoblotting using the indicated antibodies. Shown are representative images and immunoblots from N = 3 independent experiments (immunoblots depict N = 3 technical replicates in the presence of CBP). Quantification of acetylated MTs by densitometry analysis was determined **(o).** The double asterisk (**) indicates statistical significance with p-value = 0.005, as determined by student t-test.

To evaluate whether MAP acetylation can influence MT stability, MAPs were co-expressed with either wild-type CBP or an enzymatically-inactive CBP-LD mutant (L1435A/D1436A) that is incapable of acetylating MAP lysines (Fig 6). Active MAPs are known to stabilize MTs leading to prominent MT bundling or cabling, as detected by tubulin immunoreactivity [23–25]. While MT bundles still formed under all conditions tested, CBP-catalyzed acetylation of tau, MAP2c, or MAP4 (Fig 6, panels a-b, e-f, i-j) resulted in less prominent and less elaborated MT bundles compared to the baseline MAP activity observed with the inactive CBP-LD mutant (Fig 6, panels c-d, g-h, k-l), as observed by immunofluorescence with an acetylated tubulin antibody. Supporting this observation, further biochemical analysis by immunoblotting showed a reduction in either MAP2 or MAP4-mediated MT stabilization when these MAPs were acetylated in the presence of active CBP (Fig 6, panels m-o). Finally, we analyzed MAP2 in primary neurons differentiated from 4–22 days *in vitro* (DIV). Using a pan-acetyl-lysine antibody, we observed an increase in acetylated MTs (~ 55 kDa), which correlated with an apparent reduction of MAP2 acetylation-immunoreactive bands (~ 70 kDa) in mature neurons from 11–22 DIV (S2 Fig). Taken together, these results support the notion that lysine acetylation of MAPs within the MTBR can inhibit MAP-dependent MT stabilization.

## Discussion

Here, we provide the first evidence for conserved lysine acetylation as a mechanism regulating MAP2/Tau family members. Although acetylation has traditionally been viewed in the context of histones and gene transcription, recent proteomics approaches have identified acetylated targets in the cytoplasm and other organelles [15]. For example, the mitochondria harbors >100 acetylated targets encompassing 20% of total mitochondrial proteins [26], highlighting an emerging acetylation landscape. Several acetyltransferases (HATs) and deacetylases (HDACs) are enriched in the cytoplasm and are firmly associated with MTs [16, 18], suggesting that in addition to tubulin acetylation, cytosolic acetylated substrates may regulate cytoskeletal dynamics and coordinate cytoplasmic signaling events. Indeed, global proteomics analysis in cell culture and a variety of mouse tissues indicate prominent acetylation of cytoskeletal proteins including actin and microtubule-associated factors [15, 27, 28].

Our previous mass spectrometry analysis of either recombinant tau proteins or purified tau isolated from cultured cells identified four major acetylation sites in cells (K163, K280, K281, and K369) and several additional *in vitro* acetylation sites, most of which cluster within the tau MTBR [3], an observation that was recently validated and extended *in vivo* using wild-type mouse brain [7]. Since tau proteins have emerged as a dominant acetylated substrate, we investigated acetylation of the related MAP2 and MAP4 proteins, which contain sequence and structural homology [13]. Using a cell-based MAP purification method, we identified a series of acetylated MAP2 and MAP4 lysines, many of which cluster in the lysine-rich MTBR that regulates MAP-MT binding.

We reveal at least two novel acetylation motifs that show a striking conservation among MAP2 family members: [**K**-N-V-**K**/R-S-**K**] and [V-Q/**K**-I-X-X-X/**K**-**K**]. Consistent with enhanced acetylation at lysine-rich motifs, recent studies indicate that lysine acetylation preferentially occurs in a general lysine-rich local environment, in which neighboring lysines facilitate primary lysine acetylation [27, 29]. Secondly, the acetylated lysines identified in our study often contain I/V residues at positions P^+2^ or P^-2^ (e.g. ^332^VQIVT**K**K/I^339^ in MAP2c) or D/E residues at positions ranging from P^-4^ to P^+4^ (e.g. ^363^VKIESV**K/**LD^371^ in MAP2c) relative to the primary acetylated lysine. In the latter scenario, such a configuration may act to enhance local acetylation by lowering the pKa of the substrate lysine, which was recently suggested by global acetylation mapping studies [27, 30].

It is currently unclear why MAPs are redundantly acetylated at the N-terminus of repeats 2–4 within the general [V-Q/**K**-I-X-X-X/**K**-**K**] consensus acetylation motif. High resolution NMR analysis indicates that the beginning of the tau repeats represent strong binding sites for microtubules [31, 32], suggesting acetylation could provide reversible regulatory control over MAP-MT binding. While the number of repeats is important for MT stabilization (i.e. four repeats bind MTs with higher affinity than three repeats), their order may not be critical [33]. In fact, using tau proteins, one can change the connection between flanking regions and repeats, and one can even swap repeats without a major effect on tau-MT binding [33]. Thus, MAP-MT interactions could be distributed independently and spread over the entire repeat region, consistent with a weak MT binding model for tau [34]. Based on our results, it is plausible that each repeat could be regulated independently, potentially via specific acetylation events within the N-terminal [V-Q/**K**-I-X-X-X/**K**-**K**] motifs.

Tau residues K280 and K281 in the 2^nd^ repeat, in particular, make an unusually large contribution to MT-binding that is abrogated upon neutralization via lysine acetylation [3, 10]. Furthermore, both K280 and K281 can undergo acetylation individually, or in tandem (i.e. doubly acetylated), suggesting that any steric effects of individually acetylated lysines may not impair, but rather might actually promote, subsequent acetylation locally within the repeats (TJC, unpublished observations). Supporting the conservation of acetylation at tau K280/K281 in other MAPs, the homologous double lysines in the 3rd repeat of MAP2c (^332^VQIVT**KK**^338^), the 2^nd^ repeat of MAP4 (^992^VQIVS**KK**^998^) and the 3^rd^ repeat of MAP4 (^1023^VQIQN**KK**^1029^) are all targeted by acetylation, either singly or doubly, in an analogous manner (Fig 2, Tables 1 and 2). Our results support the notion of independent MT interactions among the individual repeats and are consistent with double lysine-containing motifs, in particular, contributing to strong electrostatic interactions with MTs, as originally proposed for tau [10, 33]. These are the same lysines that are also subject to acetylation, likely providing regulation of MAP function and MT dynamics.

While we and others have shown that CBP catalyzes tau acetylation with high affinity *in trans* [3, 5, 6, 21], CBP/p300 is not prominently localized to the neuronal cytoplasm where tau and related MAPs are enriched [35]. Alternatively, evidence also indicates that MAP auto-acetylation can occur in the presence of acetyl-CoA alone (Fig 3) [21]. Although this phenomenon is not fully characterized, many acetyltransferases control their own catalytic activity via positive feedback auto-acetylation [36–41]. We previously showed that tau can transfer acetyl groups onto its own lysine residues [21], a mechanism that appears to be conserved among MAP2 family members (Fig 4), which is consistent with the mechanism previously proposed for MYST and N-arylamine (NAT)-family acetyltransferases [42, 43], to which MAPs have some limited sequence homology [21]. A recent global proteomics study indicated that many acetylated substrates undergo chemical auto-acetylation in the apparent absence of acetyltransferase activity [30], in which case lysine specificity may be controlled instead by lysine accessibility, specific lysine pKa values, and the presence of basic residues preceding the acetylated lysine. Thus, the lysine-rich MTBRs, and in particular the double lysine-containing regions described here, may represent conserved sites of auto-regulated chemical acetylation once MAPs have disengaged from MTs, a PTM that would likely negatively regulate MAP-MT binding. Future studies to identify and characterize putative MAP2 family acetyltransferase(s) could further shed light on the contribution of auto-regulated chemical acetylation vs. enzyme-catalyzed acetylation within the critical MTBR domain.

In summary, we provide evidence for conservation of lysine acetylation among MAP2/Tau family proteins. Future efforts to dissect how acetylation regulates MAP conformation or binding affinity could provide insight into MT dynamics, particularly during neuronal development, neuronal plasticity, or under neurodegenerative conditions when MT regulation is particularly dynamic and vulnerable. Lastly, our study could uncover site-specific MAP acetylation at particular lysine residues (or combinations thereof) as a critical factor in disease pathogenesis that may distinguish tau from its close relatives, which could factor into the selective tau toxicity observed in AD and related tauopathies.

## Supporting Information

S1 Fig Mass spectrometry analysis confirmed MAP2c acetylation. QBI-293 cells treated were transfected with MAP2c in the presence of CBP and MAP2c was immunoprecipitated with an anti-MAP T46 antibody, separated by SDS-PAGE followed by gel excision and mass spectrometry analysis. In the absence of CBP, no acetylation was detected. In the presence of CBP, one of the major acetylated peptides identified was the doubly modified lysine containing peptide, **GGQVQIVT****KK****IDLSHVTSK** (K337/K338), with significant ion scores and p-values (see Table 1 for the full list of acetylation sites). The corresponding m/z spectrum is shown.
(TIF)

S2 Fig Acetylated MAP2 analysis in primary neuronal cultures. Primary cortical neurons were differentiated from 4–22 days *in vitro* (DIV). Neuronal lysates were harvested and analyzed by immunoblotting using pan-acetyl-lysine, MAP2, tubulin, and GAPDH antibodies. Acetyl-lysine immunoreactive protein bands correlated with MAP2 migrating bands at ~ 70 kDa (top arrow), just above the prominent ~ 55 kDa acetyl-lysine immunoreactive band that corresponds to acetylated tubulin (bottom arrow), which increased during neuronal differentiation.
(TIF)

## References

[1] Andreadis A, Brown WM, Kosik KS. Structure and novel exons of the human tau gene. Biochemistry. 1992;31(43):10626–33. 1420178 10.1021/bi00158a027

[2] Goedert M, Spillantini MG, Jakes R, Rutherford D, Crowther RA. Multiple isoforms of human microtubule-associated protein tau: sequences and localization in neurofibrillary tangles of Alzheimer's disease. Neuron. 1989;3(4):519–26. 2484340 10.1016/0896-6273(89)90210-9

[3] Cohen TJ, Guo JL, Hurtado DE, Kwong LK, Mills IP, Trojanowski JQ, et al The acetylation of tau inhibits its function and promotes pathological tau aggregation. Nature communications. 2011;2:252 10.1038/ncomms1255 21427723 PMC3120096

[4] Cook C, Carlomagno Y, Gendron TF, Dunmore J, Scheffel K, Stetler C, et al Acetylation of the KXGS motifs in tau is a critical determinant in modulation of tau aggregation and clearance. Human molecular genetics. 2014;23(1):104–16. 10.1093/hmg/ddt402 23962722 PMC3857946

[5] Kamah A, Huvent I, Cantrelle FX, Qi H, Lippens G, Landrieu I, et al Nuclear magnetic resonance analysis of the acetylation pattern of the neuronal Tau protein. Biochemistry. 2014;53(18):3020–32. 10.1021/bi500006v 24708343

[6] Min SW, Cho SH, Zhou Y, Schroeder S, Haroutunian V, Seeley WW, et al Acetylation of tau inhibits its degradation and contributes to tauopathy. Neuron. 2010;67(6):953–66. 10.1016/j.neuron.2010.08.044 20869593 PMC3035103

[7] Morris M, Knudsen GM, Maeda S, Trinidad JC, Ioanoviciu A, Burlingame AL, et al Tau post-translational modifications in wild-type and human amyloid precursor protein transgenic mice. Nature neuroscience. 2015;18(8):1183–9. 10.1038/nn.4067 26192747 PMC8049446

[8] Irwin DJ, Cohen TJ, Grossman M, Arnold SE, McCarty-Wood E, Van Deerlin VM, et al Acetylated tau neuropathology in sporadic and hereditary tauopathies. The American journal of pathology. 2013;183(2):344–51. 10.1016/j.ajpath.2013.04.025 23885714 PMC3730769

[9] Irwin DJ, Cohen TJ, Grossman M, Arnold SE, Xie SX, Lee VM, et al Acetylated tau, a novel pathological signature in Alzheimer's disease and other tauopathies. Brain. 2012;135(Pt 3):807–18. 10.1093/brain/aws013 22366796 PMC3286338

[10] Goode BL, Feinstein SC. Identification of a novel microtubule binding and assembly domain in the developmentally regulated inter-repeat region of tau. The Journal of cell biology. 1994;124(5):769–82. 8120098 10.1083/jcb.124.5.769 PMC2119949

[11] Rizzu P, Van Swieten JC, Joosse M, Hasegawa M, Stevens M, Tibben A, et al High prevalence of mutations in the microtubule-associated protein tau in a population study of frontotemporal dementia in the Netherlands. Am J Hum Genet. 1999;64(2):414–21. 10.1086/302256 9973279 PMC1377751

[12] Li W, Lee VM. Characterization of two VQIXXK motifs for tau fibrillization in vitro. Biochemistry. 2006;45(51):15692–701. 10.1021/bi061422 17176091

[13] Dehmelt L, Halpain S. The MAP2/Tau family of microtubule-associated proteins. Genome Biol. 2005;6(1):204 10.1186/gb-2004-6-1-204 15642108 PMC549057

[14] Mohan R, John A. Microtubule-associated proteins as direct crosslinkers of actin filaments and microtubules. IUBMB Life. 2015;67(6):395–403. 10.1002/iub.1384 26104829

[15] Choudhary C, Kumar C, Gnad F, Nielsen ML, Rehman M, Walther TC, et al Lysine acetylation targets protein complexes and co-regulates major cellular functions. Science. 2009;325(5942):834–40. 10.1126/science.1175371 19608861

[16] Creppe C, Malinouskaya L, Volvert ML, Gillard M, Close P, Malaise O, et al Elongator controls the migration and differentiation of cortical neurons through acetylation of alpha-tubulin. Cell. 2009;136(3):551–64. 10.1016/j.cell.2008.11.043 19185337

[17] Akella JS, Wloga D, Kim J, Starostina NG, Lyons-Abbott S, Morrissette NS, et al MEC-17 is an alpha-tubulin acetyltransferase. Nature. 2010;467(7312):218–22. 10.1038/nature09324 20829795 PMC2938957

[18] Hubbert C, Guardiola A, Shao R, Kawaguchi Y, Ito A, Nixon A, et al HDAC6 is a microtubule-associated deacetylase. Nature. 2002;417(6887):455–8. 10.1038/417455a 12024216

[19] North BJ, Marshall BL, Borra MT, Denu JM, Verdin E. The human Sir2 ortholog, SIRT2, is an NAD+-dependent tubulin deacetylase. Molecular cell. 2003;11(2):437–44. 12620231 10.1016/s1097-2765(03)00038-8

[20] Illenberger S, Drewes G, Trinczek B, Biernat J, Meyer HE, Olmsted JB, et al Phosphorylation of microtubule-associated proteins MAP2 and MAP4 by the protein kinase p110mark. Phosphorylation sites and regulation of microtubule dynamics. The Journal of biological chemistry. 1996;271(18):10834–43. 8631898 10.1074/jbc.271.18.10834

[21] Cohen TJ, Friedmann D, Hwang AW, Marmorstein R, Lee VM. The microtubule-associated tau protein has intrinsic acetyltransferase activity. Nat Struct Mol Biol. 2013;20(6):756–62. 10.1038/nsmb.2555 23624859 PMC3827724

[22] Tracy TE, Sohn PD, Minami SS, Wang C, Min SW, Li Y, et al Acetylated Tau Obstructs KIBRA-Mediated Signaling in Synaptic Plasticity and Promotes Tauopathy-Related Memory Loss. Neuron. 2016;90(2):245–60. 10.1016/j.neuron.2016.03.005 27041503 PMC4859346

[23] Burgin KE, Ludin B, Ferralli J, Matus A. Bundling of microtubules in transfected cells does not involve an autonomous dimerization site on the MAP2 molecule. Molecular biology of the cell. 1994;5(5):511–7. 7919534 10.1091/mbc.5.5.511 PMC301063

[24] Olson KR, McIntosh JR, Olmsted JB. Analysis of MAP 4 function in living cells using green fluorescent protein (GFP) chimeras. The Journal of cell biology. 1995;130(3):639–50. 7622564 10.1083/jcb.130.3.639 PMC2120526

[25] Vogelsberg-Ragaglia V, Bruce J, Richter-Landsberg C, Zhang B, Hong M, Trojanowski JQ, et al Distinct FTDP-17 missense mutations in tau produce tau aggregates and other pathological phenotypes in transfected CHO cells. Molecular biology of the cell. 2000;11(12):4093–104. 11102510 10.1091/mbc.11.12.4093 PMC15059

[26] Kim SC, Sprung R, Chen Y, Xu Y, Ball H, Pei J, et al Substrate and functional diversity of lysine acetylation revealed by a proteomics survey. Molecular cell. 2006;23(4):607–18. 10.1016/j.molcel.2006.06.026 16916647

[27] Lundby A, Lage K, Weinert BT, Bekker-Jensen DB, Secher A, Skovgaard T, et al Proteomic analysis of lysine acetylation sites in rat tissues reveals organ specificity and subcellular patterns. Cell reports. 2012;2(2):419–31. 10.1016/j.celrep.2012.07.006 22902405 PMC4103158

[28] Zhu X, Liu X, Cheng Z, Zhu J, Xu L, Wang F, et al Quantitative Analysis of Global Proteome and Lysine Acetylome Reveal the Differential Impacts of VPA and SAHA on HL60 Cells. Sci Rep. 2016;6:19926 10.1038/srep19926 26822725 PMC4731804

[29] Karabulut NP, Frishman D. Tissue-specific sequence and structural environments of lysine acetylation sites. J Struct Biol. 2015;191(1):39–48. 10.1016/j.jsb.2015.06.001 26049078

[30] Olia AS, Barker K, McCullough CE, Tang HY, Speicher DW, Qiu J, et al Nonenzymatic Protein Acetylation Detected by NAPPA Protein Arrays. ACS Chem Biol. 2015;10(9):2034–47. 10.1021/acschembio.5b00342 26083674 PMC4610810

[31] Kadavath H, Hofele RV, Biernat J, Kumar S, Tepper K, Urlaub H, et al Tau stabilizes microtubules by binding at the interface between tubulin heterodimers. Proceedings of the National Academy of Sciences of the United States of America. 2015;112(24):7501–6. 10.1073/pnas.1504081112 26034266 PMC4475932

[32] Mukrasch MD, Biernat J, von Bergen M, Griesinger C, Mandelkow E, Zweckstetter M. Sites of tau important for aggregation populate {beta}-structure and bind to microtubules and polyanions. The Journal of biological chemistry. 2005;280(26):24978–86. 10.1074/jbc.M501565200 15855160

[33] Trinczek B, Biernat J, Baumann K, Mandelkow EM, Mandelkow E. Domains of tau protein, differential phosphorylation, and dynamic instability of microtubules. Molecular biology of the cell. 1995;6(12):1887–902. 8590813 10.1091/mbc.6.12.1887 PMC366657

[34] Butner KA, Kirschner MW. Tau protein binds to microtubules through a flexible array of distributed weak sites. The Journal of cell biology. 1991;115(3):717–30. 1918161 10.1083/jcb.115.3.717 PMC2289193

[35] Jiang H, Poirier MA, Liang Y, Pei Z, Weiskittel CE, Smith WW, et al Depletion of CBP is directly linked with cellular toxicity caused by mutant huntingtin. Neurobiol Dis. 2006;23(3):543–51. 10.1016/j.nbd.2006.04.011 16766198

[36] Karanam B, Jiang L, Wang L, Kelleher NL, Cole PA. Kinetic and mass spectrometric analysis of p300 histone acetyltransferase domain autoacetylation. The Journal of biological chemistry. 2006;281(52):40292–301. 10.1074/jbc.M608813200 17065153

[37] Lu L, Li L, Lv X, Wu XS, Liu DP, Liang CC. Modulations of hMOF autoacetylation by SIRT1 regulate hMOF recruitment and activities on the chromatin. Cell Res. 2011;21(8):1182–95. 10.1038/cr.2011.71 21502975 PMC3193486

[38] Wang J, Chen J. SIRT1 regulates autoacetylation and histone acetyltransferase activity of TIP60. The Journal of biological chemistry. 2010;285(15):11458–64. 10.1074/jbc.M109.087585 20100829 PMC2857024

[39] Yang C, Wu J, Sinha SH, Neveu JM, Zheng YG. Autoacetylation of the MYST lysine acetyltransferase MOF protein. The Journal of biological chemistry. 2012;287(42):34917–26. 10.1074/jbc.M112.359356 22918831 PMC3471714

[40] Yang C, Wu J, Zheng YG. Function of the active site lysine autoacetylation in tip60 catalysis. PloS one. 2012;7(3):e32886 10.1371/journal.pone.0032886 22470428 PMC3314657

[41] Yuan H, Rossetto D, Mellert H, Dang W, Srinivasan M, Johnson J, et al MYST protein acetyltransferase activity requires active site lysine autoacetylation. EMBO J. 2012;31(1):58–70. 10.1038/emboj.2011.382 22020126 PMC3252582

[42] Dupret JM, Grant DM. Site-directed mutagenesis of recombinant human arylamine N-acetyltransferase expressed in Escherichia coli. Evidence for direct involvement of Cys68 in the catalytic mechanism of polymorphic human NAT2. The Journal of biological chemistry. 1992;267(11):7381–5. 1559981

[43] Yan Y, Harper S, Speicher DW, Marmorstein R. The catalytic mechanism of the ESA1 histone acetyltransferase involves a self-acetylated intermediate. Nat Struct Biol. 2002;9(11):862–9. 10.1038/nsb849 12368900

