# Supplementary figures and images for: Conserved Lysine Acetylation within the Microtubule-Binding Domain Regulates MAP2/Tau Family Members
Source: PLoS One. 2016 Dec 21;11(12):e0168913. doi: 10.1371/journal.pone.0168913 (PMC5176320; doi:10.1371/journal.pone.0168913)

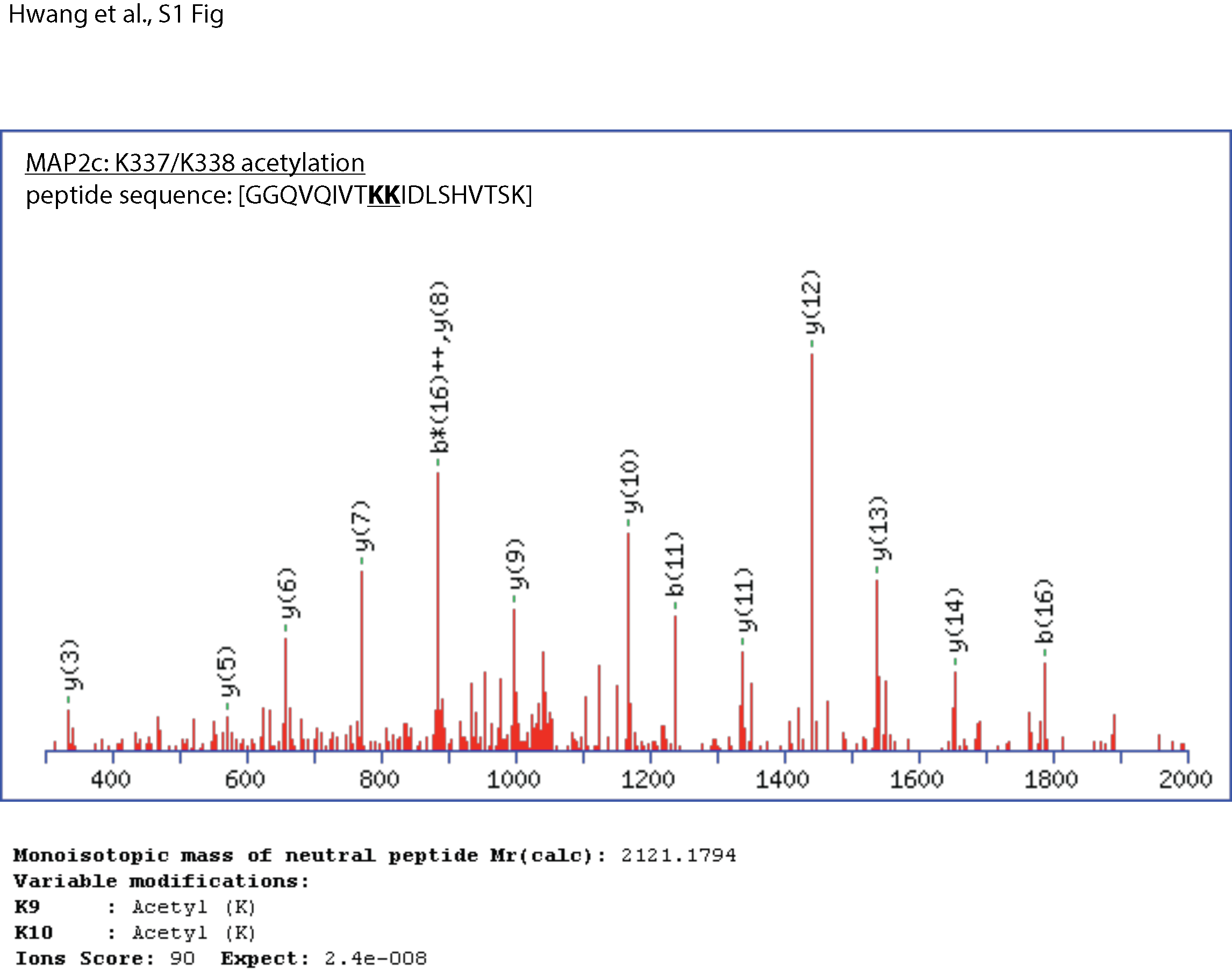

Supplement: S1 Fig — QBI-293 cells treated were transfected with MAP2c in the presence of CBP and MAP2c was immunoprecipitated with an anti-MAP T46 antibody, separated by SDS-PAGE followed by gel excision and mass spectrometry analysis. In the absence of CBP, no acetylation was detected. In the presence of CBP, one of the major acetylated peptides identified was the doubly modified lysine containing peptide, GGQVQIVTKKIDLSHVTSK (K337/K338), with significant ion scores and p-values (see Table 1 for the full list of acetylation sites). The corresponding m/z spectrum is shown. (TIF) [file pone.0168913.s001.tif]

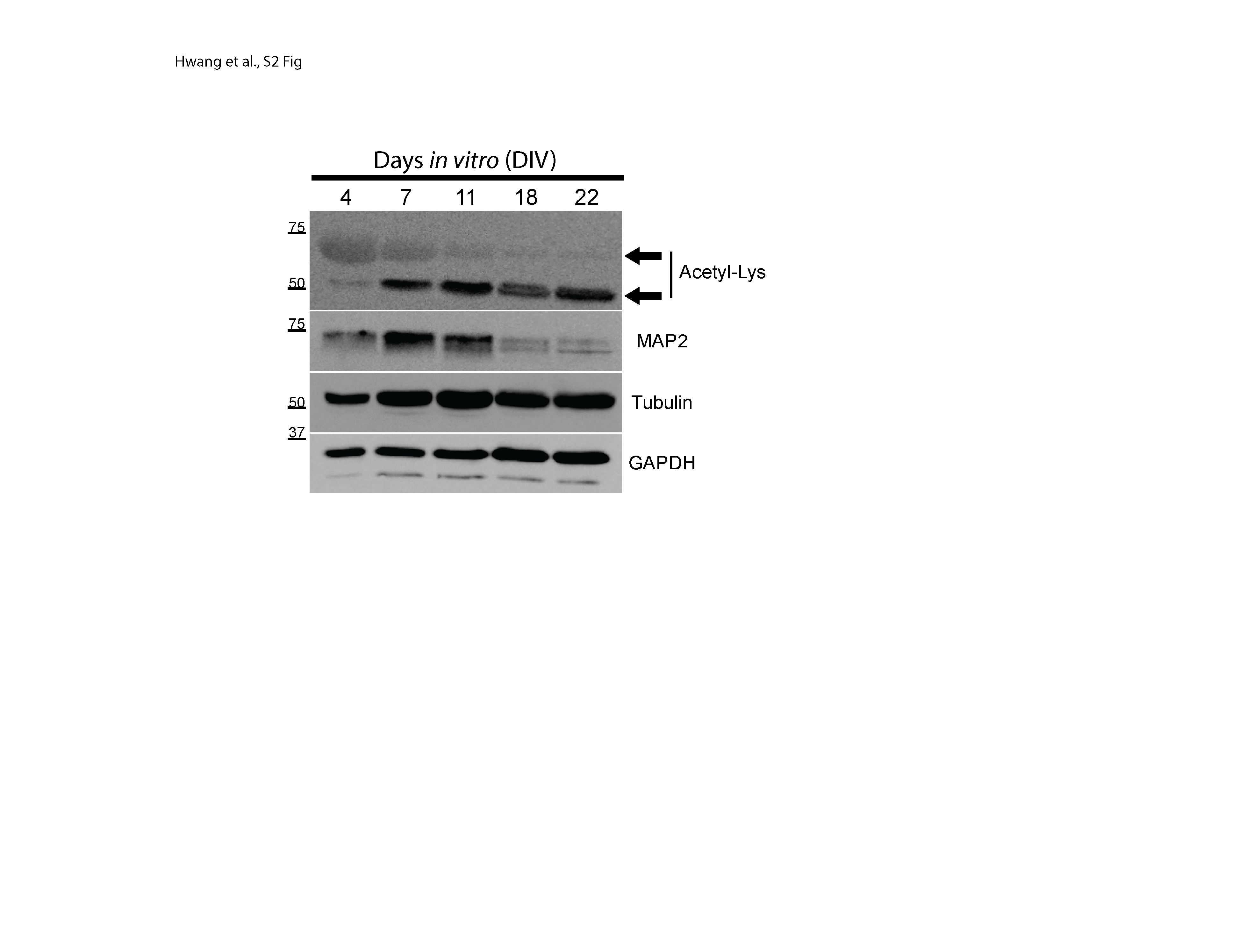

Supplement: S2 Fig — Primary cortical neurons were differentiated from 4–22 days in vitro (DIV). Neuronal lysates were harvested and analyzed by immunoblotting using pan-acetyl-lysine, MAP2, tubulin, and GAPDH antibodies. Acetyl-lysine immunoreactive protein bands correlated with MAP2 migrating bands at ~ 70 kDa (top arrow), just above the prominent ~ 55 kDa acetyl-lysine immunoreactive band that corresponds to acetylated tubulin (bottom arrow), which increased during neuronal differentiation. (TIF) [file pone.0168913.s002.tif]
